# A Panoramic X-ray as a Supportive Diagnostic Tool for the Screening of Osteoporosis in Patients with Hemophilia A and B

**DOI:** 10.3390/jcm12216901

**Published:** 2023-11-02

**Authors:** Sylwia Czajkowska, Joanna Rupa-Matysek, Kacper Nijakowski, Lidia Gil, Anna Surdacka, Tomasz Kulczyk

**Affiliations:** 1 Department of Conservative Dentistry and Endodontics, Poznan University of Medical Sciences, 60-812 Poznan, Polandannasurd@ump.edu.pl (A.S.); 2 Department of Hematology and Bone Marrow Transplantation, Poznan University of Medical Sciences, 60-812 Poznan, Poland; 3 Department of Diagnostics, Poznan University of Medical Sciences, 60-812 Poznan, Poland

**Keywords:** hemophilia A, hemophilia B, radiography, panoramic, osteoporosis, X-rays

## Abstract

Background: Hemophilia is associated with an increased risk of developing osteoporosis and osteopenia. The aim of the study was to interpret the usefulness of fractal analysis of the trabecular bone of the mandible (FD) and selected radiomorphic indices (the antegonial index (AI) and Klemetti index (KI)) to assess the risk of reduced bone mineral density (BMD) in patients with hemophilia A/B. Methods: The study group consisted of 50 patients with type A/B hemophilia. The control group consisted of 25 males without congenital bleeding disorders. The patients had a panoramic radiograph taken with the same X-ray machine (Vistapano S, Durr Dental, Bietigheim-Bissingen, Germany). The AudaXCeph software (Audax d.o.o., Ljubljana, Slovenia) was used to evaluate the AI index, and the ImageJ, software with a specially prepared script, was used to evaluate the FD. The mandibular cortex was assessed distal to the mental foramen using the Klemetti index. Results: There were no statistically significant differences between the AI, KI and FD values between the study group and the control group. Conclusions: This study indicated the lack of usefulness of AI, KI and fractal analysis in identifying patients with hemophilia at risk of reduced bone mass.

## 1. Introduction

Hemophilia is one of the most common plasma bleeding disorders. The disease, the inheritance of which is linked to the X chromosome, is characterized by a deficiency of one of the coagulation factors, actor VIII (FVIII) in the case of hemophilia A (HA) or factor IX (FIX) in the case of hemophilia B (HB). The clinical picture of both HA and HB is similar and depends on the concentration of the deficient clotting factor. A characteristic feature of hemophilia is spontaneous, excessive, or prolonged bleeding, which may affect any vascularized tissue, leading to secondary complications. It is estimated that hemophilia A occurs in 1 in 4000 to 1 in 5000 male live births, and hemophilia B occurs in approximately 1 in 15,000 to 1 in 30,000 male live births [1].

In patients with severe hemophilia (<1% of factor norm), spontaneous joint and intramuscular bleeding leads to joint destruction, development of hemophilic arthropathy and permanent disability. Moreover, patients with hemophilia have an increased risk of osteoporosis and osteopenia [2,3,4]. A decreased bone mineral density (BMD) may be associated with reduced physical activity due to intra-articular hemorrhages or fear of injury. Some authors also indicate the impact of viral infection (HBV, HCV, HIV) [2] and the direct impact of FVIII/FIX on bone metabolism [5,6,7,8]. The question remains as to the mechanism by which FVIII/FIX would influence BMD; it is possible that FVIII/FIX influences the RANK/RANKL/OPG pathway or the Wnt/β catenin pathway.

Panoramic X-ray is one of the most frequently performed examinations in patients in dental offices. It has been shown that panoramic radiographs can help assess BMD [9,10,11,12,13,14] in patients with osteoporosis and undetected low skeletal bone density. This study aims to evaluate selected parameters of the mandibular bone structure in terms of their usefulness as screening tools for assessing reduced bone mass in patients with hemophilia A or B. Based on panoramic X-ray images, the value of the fractal dimension (FD) of selected areas of cancellous bone, and indices, namely the antegonial index (AI) and Klemetti index (KI), were assessed in patients with hemophilia A and B, and a control group of healthy patients. This study is the next stage of studies previously conducted by the authors, which showed a relationship between hemophilia and the PMI (panoramic mandibular index) and no relationship with the MI (mental index) [15].

## 2. Materials and Methods

### 2.1. Study and Control Groups

The study group comprises 50 men aged 19 to 65 (median 36.5) diagnosed with mild, moderate or severe congenital hemophilia A or B, treated at the Department of Hematology and Bone Marrow Transplantation (DHBM). Oral examination was performed in 77 patients, over 70% of patients under the care of the center [16]. Radiographs were assessed in 50 patients; patients for whom it was not possible to obtain a technically correct radiograph and patients who refused to undergo radiological diagnostics were excluded from the study. The center where the research was conducted is the only center in the Greater Poland Voivodeship that implements the “National Program of Treatment of Patients with Hemophilia and Related Hemorrhagic Diathresis” for adult patients (I degree of referentiality).

The medical data of patients, retrieved from hospital files, are presented in Table 1. They include information about the type and severity of hemophilia, the routine of management, the presence of hemophilic arthropathy and other related factors.

The control group consisted of 25 males without any congenital bleeding disorders, aged 21 to 63 (median age 36.5), who also received dental treatment at the DCDE. The exclusion criteria for the control group were a history of diseases that could reduce bone mass, including parathyroid and thyroid dysfunction, chronic renal failure, steroid therapy and prolonged immobilization. Women were disqualified from the study because men suffer from hemophilia and women are carriers. Patients in the control group, like patients in the study group, lived in the Greater Poland region (Poland). The study and control groups were sex- and age-matched.

The study was conducted in accordance with the criteria of the Declaration of Helsinki. Before starting the study, the consent of the local bioethical committee and written consent for participation in the study of all patients was obtained.

### 2.2. Radiological Examination and Analysis of X-ray Images

In the course of treatment at the DCDE, patients underwent panoramic digital X-rays. All panoramic images were taken using the same X-ray machine (Vistapano S, Durr Dental, Bietigheim-Bissingen, Germany) using S-Pan’s proprietary automatic image creation technology from many parallel slices. The obtained panoramic images were saved in the DICOM (Digital Imaging and Communications in Medicine) format for further evaluation.

**Figure 1:**
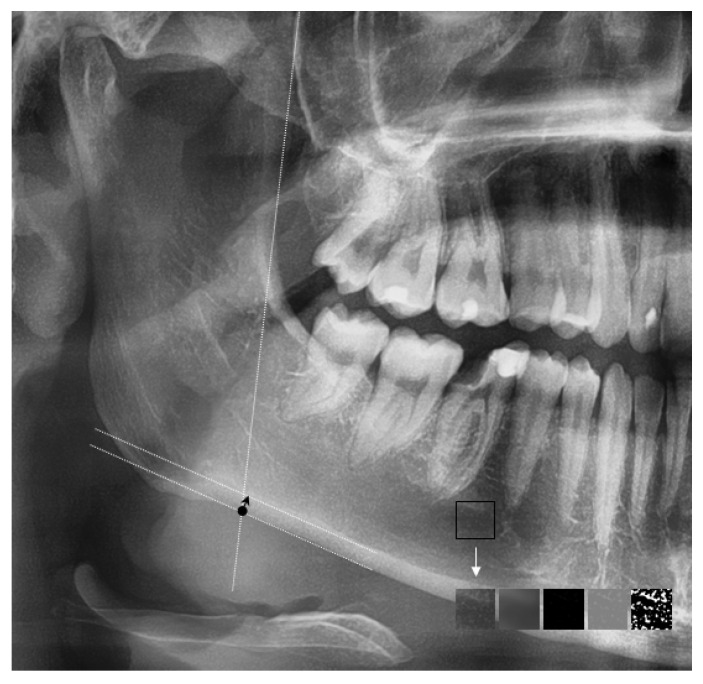
Panoramic cropped image with marked auxiliary lines to assess the thickness of the cortical plate of the lower edge of the mandible (Antagonial index—AI). Selected region of interest (ROI) enabling fractal analysis and the stages of image analysis. The black arrow—the compact bone; the white arrow—the process of transforming a region of interest into a binary version.

The antegonial index (AI) was determined and calculated employing the AudaxCeph program (Audax d.o.o., Ljubljana, Slovenia). This medical software for X-ray analysis allows the creation of customized two-dimensional measurements based on previously introduced anatomical landmarks. Within the AudaXCeph (Audax d.o.o., Ljubljana, Slovenia) software on panoramic X-ray, two lines were created for each image. The first line was parallel to the ascending ramus of the mandible (line of best fit), and the second line was parallel to the body of the mandible (Figure 1). At the intersection of the lines, the thickness of the compact plate of the lower edge of the mandible was measured.

Fractal analysis (FCA) of the trabecular bone of selected regions of the mandible was performed using the ImageJ version 1.53k software (National Institutes of Health, Public Domain), employing the method described by White and Rudolph [17]. The selected regions of interest (ROI) of 60 × 60 pixels, located distally to the mental foramen, above the margin of the cortex and below the tips of the roots, were cropped from the X-ray image and stored for detailed further analysis (Figure 1). The above area was selected due to the lack of overlapping anatomical structures, such as the cervical spine, maxillary sinus or air between the hard palate and the base of the tongue. According to the authors, the selected anatomical location is the most reliable place for assessing cancellous bone. When selecting the ROI, locations that could represent a periapical lesion were avoided. To perform FCA, the given ROIs were converted into binary images in a series of modifications. At first, a Gaussian 5-pixel filter was applied to create a blurred version of the ROI’s image. Then, the blurred image was subtracted from the original image and normalized by setting the mean intensity at a value of 128. Finally, the normalized image was converted into a binary image. The fractal dimension of the binary image of the ROI was calculated by means of the FracLac plugin in the ImageJ software.

The Klemetti index (KI) of the cortical bone of the mandible was assessed in the region distal to the mental foramen using the C1, C2 and C3 scale. The C1 index was set when the inner margin of the cortical bone was smooth and continuous on the right and left sides. The C2 index was set when the inner edge of the cortical bone depicted intraosseous defects or lacunar resorption on one or both sides. The C3 index was set when the inner margin of the cortical bone was visibly porous and permeable.

The KI index assessment was performed by one observer. Three months after the completion of the measurements, 10 randomly selected radiological panoramic images were re-analyzed to calculate the intraclass correlation coefficient (ICC). The achieved ICC value was 0.9392.

## 3. Results

There were no statistically significant differences between the values of the AI, KI and FD (Table 2 and Table 3) between the study group (hemophilia) and the control group.

The severity of hemophilia (mild, moderate, severe) did not affect the selected radiomorphic indices or fractal analysis (Table 4). The occurrence of hemophilic arthropathy did not affect the values of the studied indicators (Table 5). There was no significant difference in the AI and FDs depending on the antihemorrhagic therapy used (Table 6).

## 4. Discussion

The above analyses are a continuation of studies published by the authors of this paper. It is based on the assumption that the X-ray image of the maxillofacial area in patients with HA and HB may show certain detectable features which are different from those for healthy individuals. These differences can be detected using indexes.

We previously demonstrated the usefulness of the PMI (panoramic mandibular index) in the diagnosis of reduced bonein patients with congenital hemophilia. We reported that the PMI, combined with the concentration of citrullinated histone H3 (CH3) and osteocalcin (BGLAP), may help identify patients with congenital hemophilia requiring additional diagnostics for reduced bone mass. Also, a previous study showed no correlation between hemophilia and bone turnover in the case of MI (mental index). This continuation of the conducted research is the inclusion of additional radiomorphometric indices in the experiment: the antegonial index (AI) and Klemetti index (KI) and the use of fractal dimension analysis (FD). The AI, expressed in mm, is an indicator of the thickness of compact bone in front of the gonion landmark in the mandible.

KI is a qualitative indicator based on the appearance of the mandibular cortex distally to the mental foramen. Fractal dimension analysis was employed as the bone tissue pattern has a fractal structure.

When selecting the area for evaluation, we paid particular attention to ensure that it was located within the cancellous bone. The ROI did not overlap with the radiographic image of the teeth. Due to the numerous anatomical structures within the craniofacial region, it is very difficult to select the ideal area for ROI assessment. In the anterior section of the maxilla and mandible, assessment is made impossible by the overlapping image of the cervical spine. The lateral section of the maxilla is not suitable for evaluation due to the presence of the right and left maxillary sinuses and the air between the base of the tongue and the hard palate. In addition, there is an inferior alveolar nerve canal in the mandible, which should also not be located within the ROI. The authors are aware of the possibility of Stafne’s bone cavity (SBC) occurring in the mandible. According to the latest research, SBC occurs with a frequency of 0.17% [18].Taking the above into account, the authors selected the safest area for evaluation. Of course, the presence of a periapical lesion disqualified a given area from evaluation. The study showed no statistically significant differences in the values of AI, KI or FD between the study group and the control group. We concluded that the above indicators are not helpful in quickly identifying patients with HA/HB with low bone mass.

However, we want to point out that in the study group, only 20% of patients had a sufficient level of vitamin D. Also, as many as 20% of patients had serum calcium levels above the norm, indicating a potential risk of bone turnover disorders in the study group. An increased risk of osteopenia and osteoporosis is also suggested by the diagnosis of hemophilic arthropathy in 68% of the subjects.

Many authors reported that radiographic features in the maxillofacial region might be associated with systemic disease, including hematologic disease. So far, the possibility of using FD in assessing the mandible bone in panoramic radiographs in patients with sickle cell anemia and thalassemia has been proven [19]. Serindere and Belgin analyzed 90 panoramic images, thus showing that the FD values in the interproximal area are higher in patients with thalassemia compared to healthy controls. The results of the analysis may suggest the need to extend the research to other hematological diseases that may affect the skeletal system. In their study, Serindere and Belginassessed the fractal bone dimensions (FD) and used selected radiomorphic indices. In patients with hemoglobinopathy, the PMI and MCW (mandibular cortical width) values are significantly higher in the control group than in the study group. Even though the research results differed from ours, which may be related to a specific disease entity, the researchers emphasized dental radiology’s role in assessing bones in hematological diseases.

Besides the study of Serindere and Belgin, Neves et al. attempted to find a relationship between the severity of sickle cell anemia and the radiographic features observed in panoramic X-rays [20]. In this study, medical data such as incidence of vasomotor crisis, history of stroke, episodes of jaundice, femoral head necrosis and occurrence of lower limb ulcers were combined with data obtained from panoramic images. Researchers showed a statistically significant relationship between increased trabecular spacing and episodes of jaundice and between the presence of horizontal trabeculae and a history of stroke. The authors concluded that the clinical parameters of systemic complications might affect the radiographic features in the maxillofacial area.

Initially, radiomorphometric indices were employed in the assessment of bone mass in postmenopausal women [10,11,21]. However, Dagistan et al. proved that radiomorphic indices, including the MI, PMI and AI, can be successfully used as a tool helpful in the diagnosis of osteoporosis also in males [9].

Researchers used DXA to assess bone mass in the lumbar region (L1–L4) and proximal femur in 40 males. The obtained results were correlated with the values of radiomorphometric indices, thus indicating the usefulness of dental radiology in diagnosing osteoporosis in males. The authors emphasized the use of the MI, PMI and AI, and also stated the need for further research to assess the usefulness of the Klemetti index (in the study, described as the mandibular cortical index—MCI).

The study of Dagistan and associates is critical in patients with hemophilia, as the examined bleeding disorder is inherited in an X-linked recessive manner.

Due to the presence of two X chromosomes, females suffer from hemophilia only in exceptional situations.

An example is a situation of extreme lionization or in the case of a chromosomal aberration, which includes, among others, monosomy of the X chromosome [22].

The survey by Dagistan and associates showed no statistically significant difference in the MCI value in the study and control groups.

The authors of this experiment emphasize that the primary diagnostic test used to assess bone mineral density is DXA (double energy X-ray absorptiometry); however, one should not forget the role of dental radiology in diagnosing reduced bone mass. Several papers confirm the usefulness of orthopantomographic images to identify patients with low bone mass, which was approved, among others, in the OSTEODENT project [23,24,25,26,27,28]. In this multi-center study, researchers used more than 600 postmenopausal women’s data, thus diagnosing osteoporosis in 140 women. One of the goals of the OSTEODENT project was to show whether panoramic images could be an additional screening tool for identifying which patients should undergo DXA.

Studies by Wallny et al. [2] and Mansouritorghabeh et al. [3] proved that patients with congenital hemophilia develop bone metabolism disorders, which results in an increased risk of osteopenia and osteoporosis.

Wallny et al. [2] assessed bone mineral density (BMD) in patients with severe hemophilia type A using DXA, and combined it with the clinical score of the severity of hemophilic arthropathy (which was diagnosed in 88.7% of the study group patients). In this study, osteopenia was found in 43.5% and osteoporosis in 25.8% of subjects (examinations involved the femoral neck and/or lumbar spine). Wallny et al. [2] emphasize that the correlation of the BMD with the examined variables of the femoral neck showed a better correlation than of the BMD with the lumbar spine variables. In addition, the researchers pointed to additional risk factors for reduced BMD: a history of hepatitis C, age, low BMI and the number of joints with hemophilic arthropathy.

Some studies showed reduced bone mass in patients with hemophilia type B [3,29]. In the study by Mansouritorghabeh et al., decreased BMD occurred in the L2–L4 region and femur [3]. In addition, the authors indicated a high frequency of hepatitis C infection, but the data were insufficient to assess the impact of infection on BMD. A weakness of the study was the small size of the study group. Similar results to those of Wallny et al. and Mansouritorghabeh et al. were reported by Sahina et al. [29]. The authors assessed the bone mineral density in 61 patients aged 20 to 63 (median 37.8) with hemophilia A and B. The researchers noted the occurrence of reduced bone mineral density in as many as 49.15% of patients while emphasizing that the severity of hemophilia, the average annual number of bleeds or the anti-bleeding therapy used (factor on-demand prophylactically) have no effect on BMD. Sahin et al. also reported that as many as 79.31% of young patients with hemophilia had a significantly reduced vitamin D level (<20 ng/mL), which is consistent with our observations.

## 5. Conclusions

This study demonstrates that the AI and KI indices and FD analysis of bone patterns derived from panoramic dental X-ray images do not help identify patients with hemophilia at risk of reduced bone mass. Our study indicated a deficiency of, or insufficient, vitamin D levels in 80% of patients with hemophilia, and elevated serum calcium levels in 20% of patients.

## Figures and Tables

**Table 1:** Characteristics of study group.

		*n*	%	Age	
The Study Group				Median	Age Range
**Type of hemophilia**	A	40	80	36	19–65
	B	10	20	41.5	26–56
**Routine management**	On-demand therapy	22	44	33.5	20–65
	Secondary prophylactic therapy	28	56	38.5	19–62
**Severity of hemophilia**	Severe	35	70	40	19–62
	Moderate	7	14	32	26–56
	Mild	8	16	24.5	20–65
**Hemophilic arthropathy**	Yes	34	68	41	22–62
	No	16	32	27	19–65
**Ca serum level**	Elevated	10	20.8	35.5	26–46
	Normal	38	79.2	40.5	19–65
**Vitamin D serum level**	H < 20.0 deficiency	25	51	35	20–62
	20–30 insufficient level	14	28.6	37	19–65
	L 30–100 sufficient level	10	20.4	46.0	31–58
**Smoking**	Yes	17	34	41	21–58
	No	33	66	35	19–65

**Table 2:** Comparison of AI and FD between the study group and the control group.

	Study Group		Control Group		*p*-Value
	*n* = 50		*n* = 25		
	M [Q1–Q3]	Min–Max	M [Q1–Q3]	Min–Max	
AI	1.80 [1.56–2.04]	1.08–3.55	1.92 [1.71–2.17]	1.46–3.22	0.055
FD	1.55 [1.51–1.60]	1.42–1.65	1.54 [1.51–1.58]	1.43–1.63	0.525

*p*-value < 0.05 for the Mann–Whitney test. AI—antegonial index; FD—fractal analysis, Min—minimum value; Max—maximum value; M—median; Q1—lower quartile, Q3—upper quartile.

**Table 3:** Comparison of Klemetti index values for the study group and the control group.

	C1	C2	C3
**Study group**	6	39	5
**Control group**	5	20	0

Pearson’s chi-square test: *p*-value = 0.198.

**Table 4:** Comparison of AI and FD depending on the severity of hemophilia—mild and moderate hemophilia and severe hemophilia.

	Mild + Moderate		Severe		*p*-Value
	*n* = 15		*n* = 15		
	M [Q1–Q3]	Min–Max	M [Q1–Q3]	Min–Max	
AI	1.79 [1.57–1.93]	1.32–2.18	1.81 [1.53–2.05]	1.08–3.55	0.966
FD	1.55 [1.52–1.58]	1.48–1.61	1.56 [1.49–1.61]	1.42–1.65	0.498

AI—antegonial index; FD—fractal analysis, Min—minimum value; Max—maximum value; M—median; Q1—lower quartile, Q3—upper quartile.

**Table 5:** Comparison of AI and FD depending on the anti-hemorrhagic therapy used—factor administered “on demand” or factor administered prophylactically.

	On-Demand Therapy		Prophylactic Therapy		*p*-Value
	*n* = 22		*n* = 28		
	M [Q1–Q3]	Min–Max	M [Q1–Q3]	Min–Max	
AI	1.78 [1.53–1.93]	1.08–2.18	1.83 [1.60–2.07]	1.09–3.55	0.353
FD	1.55 [1.52–1.59]	1.47–1.62	1.54 [1.49–1.60]	1.42–1.65	0.837

AI—antegonial index; FD—fractal analysis, Min—minimum value; Max—maximum value; M—median; Q1—lowerquartile, Q3—upper quartile.

**Table 6:** Comparison of AI and FD between patients with and without hemophilic arthropathy.

	With Arthropathy		Without Arthropathy		*p*-Value
	*n* = 34		*n* = 16		
	M [Q1–Q3]	Min–Max	M [Q1–Q3]	Min–Max	
AI	1.84 [1.48–2.05]	1.08–3.55	1.74 [1.65–1.91]	1.32–2.12	0.610
FD	1.54 [1.49–1.60]	1.42–1.65	1.57 [1.52–1.59]	1.43–1.62	0.716

AI—Antegonial index; FD—fractal analysis, Min—minimum value; Max—maximum value; M—median; Q1—lower quartile, Q3—upper quartile.

## Data Availability

The data presented in this study are available on reasonable, common-sense request from the corresponding author.
